# Are Fibromyalgia and Obsessive‐Compulsive Personality Disorder related to Lewy Body Dementia?

**DOI:** 10.1002/alz70857_106955

**Published:** 2025-12-25

**Authors:** Maria Cecilia Fernández, Waleska Berrios, Roni Eichel, Federico Rodriguez‐Porcel, Angel Golimstok

**Affiliations:** ^1^ Hospital Italiano de Buenos Aires, Buenos aires, Argentina; ^2^ Shaare Zedek Medical Center, Jerusalem, Israel; ^3^ Medical University of South Carolina, Charleston, SC, USA

## Abstract

**Background:**

Recent studies suggest a potential link between attention‐deficit/hyperactivity disorder (ADHD) and the development of Lewy body disease (LBD). Fibromyalgia and obsessive‐compulsive personality disorder (OCPD) have been associated with ADHD in adults. This study aimed to determine the extent to which the presence of these two conditions increases the risk of LBD or any of its clinical features.

**Method:**

This prospective cohort study followed participants over a 15‐year period. The study compared a group of adults diagnosed with ADHD, meeting DSM‐IV‐TR criteria, with a healthy control group. All participants were evaluated at baseline and follow‐up for the presence of fibromyalgia (according to the American College of Rheumatology criteria) and OCPD (based on DSM criteria). Univariate analyses and multiple logistic regression models were used to examine the association between fibromyalgia, OCPD, and LBD features, adjusting for multiple variables.

**Result:**

The baseline sample included 161 individuals with ADHD and 109 without ADHD. After the 15‐year follow‐up, 31 participants developed dementia, with 27 cases in the ADHD group and 4 in the control group. LBD was the most common type (*N* = 20), with 19 cases occurring in the ADHD group. LBD features were significantly more prevalent in the ADHD group, the most frequent being cognitive fluctuations and REM sleep behavior disorder (RBD). Multivariate logistic regression analysis showed that individuals with fibromyalgia had increased odds of developing certain LBD features, including cognitive fluctuations (OR: 1.46, 95% CI: 0.6–3.57), RBD (OR: 1.29, 95% CI: 0.56–3.02), visuospatial dysfunction (OR: 1.56, 95% CI: 0.65–3.73), and depression (OR: 2.25, 95% CI: 0.89–5.7). The presence of OCPD was associated with higher odds of fibromyalgia (OR: 3.73, 95% CI: 1.59–8.8) and depression (OR: 1.6, 95% CI: 0.88–2.88) but was not related to other LBD features.

**Conclusion:**

Fibromyalgia in individuals with ADHD may increase the risk of developing certain features of LBD, including cognitive fluctuations, RBD, visuospatial dysfunction, and depression. Future research is needed to further investigate these findings and elucidate the underlying pathophysiological mechanisms.

